# Editorial: Machine vision and machine learning for plant phenotyping and precision agriculture

**DOI:** 10.3389/fpls.2023.1331918

**Published:** 2023-11-27

**Authors:** Huajian Liu, Zhanyou Xu

**Affiliations:** ^1^ Australian Plant Phenomics Facility, The Plant Accelerator, School of Agriculture, Food and Wine, University of Adelaide, Urrbrae, SA, Australia; ^2^ United States Department of Agriculture – Agricultural Research Service - Plant Science Research, St. Paul, MN, United States

**Keywords:** plant phenotyping, precision agriculture, machine vision, machine learning, deep learning

Plant phenotyping (PP) describes the physiological and biochemical properties of plants affected by both genotypes and environments. It is an emerging research field assisting the breeding and cultivation of new crop varieties to be more productive and resilient to challenging environments. Precision agriculture (PA) uses sensing technologies to observe crops and then manages them optimally to ensure that they grow in healthy conditions, have maximum productivity, and have minimal adverse effects on the environment. Traditionally, the observation of plant traits heavily relies on human experts, which is labour-intensive, time-consuming, and subjective. Although PP and PA are two different fields, they share similar sensing and data processing technologies in many respects. Recently, driven by computer and sensor technologies, machine vision (MV) and machine learning (ML) have contributed to accurate, high-throughput and nondestructive sensing and data processing technologies to PP and PA. However, these technologies are still in their infant stage, and many challenges and questions related to them still need to be addressed.

This Research Topic aims to share the latest research results on applying MV and ML to PP and PA. It demonstrates cutting-edge technologies, bottle-necks and future research directions for MV and ML in crop breeding, crop cultivation, and disease or pest management. This Research Topic of Frontiers in Plant Sciences published a total of 28 peer-reviewed research articles, including one review paper for the phenotyping of Prunoideae fruits (Liu et al.). These articles reveal the latest research trends regarding different crop species, data types and algorithms.

The summary of the published reports shows that cotton (*Gossypium*), canola or oilseed rape (*Brassica napus*), wheat (*Triticum*) and maize (*Z. mays*) are the most important crops for study in PP and PA (**Figure 1A**). Cotton stands out as the most frequently examined crop, with a total of five articles dedicated to it. Yan et al. developed a leaf segmentation method in the field environments. Tang et al. investigated early detection of Verticillium wilt disease in roots. Huang et al. studied the automatic segmentation technique of roots in soil. Li et al. unveiled the evolutionary history of cotton seeds through the morphological structure of the seeds. Lastly, She et al. proposed a detection and counting method of pigment glands in cotton leaves. Canola, also known as oilseed rape, is the subject of four articles, each addressing distinct aspects of research. These include the automatic counting of inflorescences in the field environment using a UAV (Li et al.), phenotyping mature pods (Corcoran et al.), the segmentation of siliques of individual plants (Qiao et al.) and the measurement of leaf area in a laboratory environment (Li et al.). In the realm of wheat research, three pivotal studies have emerged. These encompass real-time determination of the flowering period for field wheat by detecting florets and spikelets (Song et al.), the estimation of wheat tiller density using remote sensing data (Hu et al.) and the assessment of wheat stripe rust disease severity by measuring individual leaves (Jiang et al.). Three noteworthy manuscripts, centred around maize research, have been published. These manuscripts encompass the identification of the inter-row environment during the middle and late stages for robotic navigation (Li et al.), emergence timing detection (Das et al.) and the segmentation and classification of maize seeds (Dong et al.). Other research in the main crops includes rice seedling growth traits detection (Ye et al.) and soybean canopy features description called canopy fingerprints (Young et al.). Studies focusing on vegetables or fruits have been conducted with a range of objectives. These include the estimation of anthocyanin concentrations in lettuce (Kim and Iersel), the early detection of plant stress of lettuce (Qin et al.), tomato detection (Mbouembe et al.) and the yield estimation of longan based on UAV images (Li et al.). An interesting avenue of research delves into the phenotyping of traditional Chinese herbs. Xu et al. developed a non-destructive classification method for *Astragalus membranaceus* var. *mongholicus*, *Astragalus membranaceus* and similar seeds; Zhao et al. studied the early detection of ginseng root diseases through leaves. Jung et al. investigated the classification method of *Cynanchum wilfordii* and *Cynanchum auriculatum*. Finally, Wang et al. proposed a technique for segmenting overlapped tobacco shred images and calculating their respective areas. In addition to the above-mentioned plants, two notable papers encompass the phenotyping of multiple plant species. One is about the tiller estimation method of grass plants (Kinose et al.) and another is about multiple plant leaf disease identification, providing valuable insights for disease management in various plants (Chen et al.).

**Figure 1:**
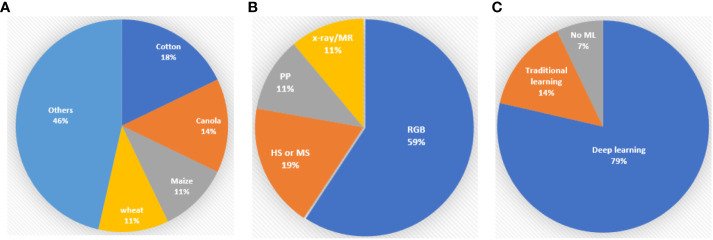
A summary of the percentage of crops, data types and machine learning algorithms in the Research Topic. PP stands for Point cloud, HS for hyperspectral data, MS for multispectral data, MR for magnetic resonance data, RGB for red-green-blue images and ML for machine learning algorithms. **(A)** Percentage of crops. **(B)** Percentage of data types. **(C)** Percentage of Machine learning algorithms.

Regarding data type, red-green-blue (RGB) images are the most commonly employed in PP and PA (**Figure 1B**) due to their cost-effectiveness and the availability of diverse algorithms. RGB images have proven to be particularly suitable for leaf segmentation (Yan et al.), tiller counting (Kinose et al.) and fruit counting (Li et al.; Mbouembe et al.). Although hyperspectral or multispectral technologies have shown great advantages over RGB imaging in PP and PA (Bruning et al., 2020; Liu et al., 2020a; Xie et al., 2021), it has not been adequately investigated. Within the scope of this Research Topic, only five publications delve into hyperspectral technologies. Xu et al. used hyperspectral imaging technologies to classify *Astragalus membranaceus* var. *mongholicus*, *Astragalus membranaceus* and similar seeds which is challenging in RGB images. Zhao et al. successfully applied hyperspectral reflectance of ginseng leaves to do early detection of root diseases. To estimate anthocyanin concentrations in lettuce at the canopy scale, Kim and Iersel used a multispectral imaging technique to develop a vegetation index called normalized difference anthocyanin index (NDAI), which is based on the optical properties of anthocyanins. Qin et al. developed a hyperspectral imaging system for plant health monitoring in a controlled-environment at NASA Kennedy Space Centre. It can conduct early detection of drought stress of twelve Dragoon lettuce samples when there are no visible symptoms. Hu et al. successfully applied hyperspectral and multispectral remote sensing data to tiller density estimation and this method could be applied to plot and county scale. LiDAR or RGB-image generated 3D point clouds demonstrated the capability to detect detailed morphological features of individual plant structures of oilseed rape (Qiao et al.), canopy structures of soybean (Young et al.) and inter-row information of maize crops (Li et al.). Micro-CT technologies showed advantages in seed phenotyping (Li et al.; Corcoran et al.) and magnetic resonance was suitable for detecting root features (Tang et al.).

This Research Topic underscores the pivotal role that machine learning, particularly deep learning, plays in PP and PA (**Figure 1C**). Deep learning algorithms, which are founded on artificial neural networks with multiple layers, feature prominently. Among the 28 collected articles, a substantial 22 of them incorporate deep learning algorithms. Notably, You Only Look Once (YOLO), U-net, and the attention mechanism emerge as the most frequently employed techniques. YOLO is an object detection algorithm based on convolutional neural network (CNN) architecture. It was first introduced by Redmon et al. (2016) and it has evolved over time with seven versions. YOLO has become popular because of its speed and ability to detect multiple objects in real-time. Five studies in this research domain have employed YOLO, encompassing detecting rapeseed inflorescences (Li et al.), inter-row environment identification of maize fields (Li et al.), tomato detection (Mbouembe et al.), counting the florets and spikelets of wheat (Song et al.) and estimating the yield of longan fruit (Li et al.). U-Net was introduced by Ronneberger et al. (2015) and it is a CNN architecture specifically designed for image segmentation tasks in computer vision. In this Research Topic, it was used for several segmentation tasks including cotton roots system in magnetic resonance images (Tang et al.), cotton seeds in micro-CT images (Li et al.), pigment glands in cotton leaves in RGB images (She et al.), coleoptile of maize in RGB images (Das et al.) and seeds of oilseed rape in micro-CT images (Corcoran et al.). The concept of attention models originally stemmed from sequence-to-sequence learning, as proposed by Sutskever et al. (2014). Over time, it has undergone several iterations and versions, adapting and evolving to suit various applications and challenges in the field of deep learning. It allows a neural network to focus on specific parts of input data while processing it. One of the key early papers that popularized attention mechanisms in deep learning is “Attention is all you need” by Vaswani et al. (2023). In the study of Huang et al. in order to reduce the influence of the background noise of cotton roots in minirhizotron images, they integrated a global attention module into object-contextual representation net (OCRNet) (Yuan et al., 2021) to enhance the focus of the model on the root targets. Li et al. applied an attention module to U-net to improve the segmentation of leaves of oilseed rape. The review article of Liu et al. also demonstrates that YOLO with the convolutional block attention module is a hot Research Topic in PP and PA. Four studies used traditional machine learning methods and all of them for hyperspectral data processing. Xu et al. used a support vector machine algorithm to classify *Astragalus membranaceus* var. *mongholicus*, *Astragalus membranaceus* and similar seeds. Zhao et al. adopted the random forest algorithm to detect ginseng root diseases early. Qin et al. investigated a discriminant classifier for the estimation of draught stress of lettuce. Hu et al. proposed to use a gradient-boosted regression tree and random forest to estimate wheat tiller density. For details of the traditional machine learning algorithms, readers can refer to the Sciket-Learn toolbox (ScikitLearn, 2023).

In summary, this Research Topic has compiled the most recent research articles in the domains of PP and PA. It not only disseminates the latest technologies, methods, and findings in PP and PA but also sheds light on the research trajectories and future directions within these fields. The Research Topic highlights that cotton, canola or oilseed rape, wheat and maize are the most extensively studied crops in the field of PP and PA. RGB cameras are the most commonly used sensors because of the lower cost, ease of operation and algorithms available. Although hyperspectral or multispectral images contain more information than RGB images and reveal more complex traits of plants, they have not been widely used. This is because of the higher system cost and the challenges of operation, especially in the field environment (Liu et al., 2020b). Machine learning is undeniably a pivotal component of PP and PA. Currently, the prevailing research trend involves the fusion of RGB imaging with deep learning techniques. Traditional machine learning methods continue to have significant relevance, particularly in hyperspectral data processing. However, it’s worth highlighting that deep learning has not yet received extensive exploration in the realm of hyperspectral data processing. Indeed, most deep learning algorithms have their origins in RGB images, and the transition to applying them to hyperspectral images can be challenging due to the differences in spatial and spectral dimensions. However, this challenge also serves as a catalyst for a promising new research direction, where innovative approaches and techniques are required to effectively adapt and apply deep learning to hyperspectral images. The bottleneck limiting the broader application of deep learning in the agricultural industry stems from insufficient training data, as well as the substantial workload and high costs associated with manual annotation, as discussed by Dong et al. (2023). Additionally, Tang et al. highlighted the challenges of data imbalance. To address these obstacles and pave the way for more extensive deep learning use in agriculture, future research directions should encompass data augmentation techniques, self-supervised learning methods, and the generation of synthetic training data.

## Author contributions

HL: Conceptualization, Formal Analysis, Project administration, Writing – original draft. ZX: Formal Analysis, Project administration, Writing – review & editing.
